# How the Closure of a U.S. Tax Loophole May Affect Investor Portfolios

**DOI:** 10.3390/jrfm15050209

**Published:** 2022-05-04

**Authors:** Christoph Frei, Liam Welsh

**Affiliations:** 1 Department of Mathematical and Statistical Sciences, University of Alberta, Edmonton, AB T6G 2G1, Canada; 2 Department of Statistical Sciences, University of Toronto, Toronto, ON M5G 1Z5, Canada; liam.welsh@mail.utoronto.ca

**Keywords:** portfolio allocation, ETFs, mutual funds, capital gains tax

## Abstract

In the United States, exchange-traded funds can defer capital gains taxes of their investors by taking advantage of a legal loophole. To quantify the impact of this tax loophole on investor portfolios, we study a rank-dependent expected utility model. We develop an approximation formula for the sensitivity of the optimal investment strategy with respect to changes in the expected asset returns. By applying this approximation formula, we are able to quantitatively estimate how much investor portfolios may change depending on the investment horizon if the tax loophole is closed.

## 1. Introduction

Exchange-traded funds (ETFs) have experienced a meteoric rise in popularity since their inception, largely due to their lower costs compared to mutual funds (MFs) and high tax efficiency. Particularly, ETFs in the United States are able to take advantage of a tax loophole to avoid capital gains distributions. In the United States, tax typically must be paid on capital gains distributions. However, due to a 1969 tax law, ETFs are able to make use of tax-free in-kind redemptions to allow their investors to defer their capital gains taxes to a future date. This causes the ETF to have a greater unrealized return for holding investors. There has been recent attention, from both media (Mider et al. 2019) and academia (Colon 2017; Hodaszy 2016; Moussawi et al. 2021) on the legal and financial aspects of this benefit for ETF investors. In September 2021, U.S. Senate Finance Committee Chairman Ron Wyden released draft legislation that could mean the beginning of the end of the ETF tax loophole although the proposal is not yet part of any formal legislative plan (Lim and Rubin 2021).

The objective of this paper is to provide a quantitative analysis of how investor portfolios containing ETFs and MFs would change in the event the ETF tax loophole is closed, in addition to providing a further understanding of how investor preferences and market components would affect this change. To this end, we develop an approximation formula that describes how an investor’s utility and their optimal asset allocation change when the expected asset returns shift. We do this in a simple model, yet capture a crucial feature in asset allocation, namely, the investors’ overweighting of tail probabilities. Compared to results from classical portfolio optimization, there exists an investor preference for actively managed mutual funds over their passively managed counterparts (e.g. ETFs), which is referred to as the mutual fund puzzle (Gruber 1996). Therefore, we use a rank-dependent expected utility (RDEU) model, as presented in Quiggin (1993). RDEU is an extension of classical expected utility that overweights unlikely extreme outcomes.

The ETF tax loophole is the unexpected outcome of a 1969 U.S. tax law and predates the first ETF by two decades (Colon 2017). For a detailed description of how the loophole is used, we refer the reader to Colon (2017); Mider et al. (2019); or Moussawi et al. (2021). The ethical considerations of this loophole, as well as its implications and possible closure scenarios, have been thoroughly discussed in the academic literature, including by Hodaszy (2016), Colon (2017), and Moussawi et al. (2021). While Hodaszy (2016) entertains various closure scenarios, Colon (2017) argues that the tax loophole provides an unfair tax benefit to ETFs and their investors, particularly, high-net-worth taxpayers. Moussawi et al. (2021) thoroughly explore in an empirical analysis the implications and results from the tax loophole. Specifically, they find that the loophole is causing an increase in capital flow from active MFs to ETFs, and as ETF investors can defer the realization of their capital gains to an optimal future date due to the tax loophole, ETFs are becoming a more popular investment with high-net-worth investors.

The remainder of this paper is organized as follows. In Section 2, we form an approximation on the RDEU portfolio optimization problem under changes in the expected asset returns. In Section 3, we estimate the benefits ETF investors receive due to the tax loophole, analyze how an investor’s portfolio would change in the event the loophole is closed, and discuss the sensitivities of this investment change with respect to the parameter values. Finally, in Section 4, we provide our closing remarks. All proofs are relegated to Appendix A.

## 2. Approximation Formula

We consider a financial market with *T* trading periods. There are *n* risky assets in addition to a risk-free asset with interest rate $r_{f}$. The returns of the risky assets in period *t* are represented by a random vector $Y_{t}$ with *n* components. We assume that the return vectors ${\left(Y_{t}\right)}_{t=1,\cdots ,T}$ are independent for different periods, but they do not need to have independent components. The expected return vector and covariance matrix of $Y_{t}$ are denoted by $\mu_{t}$ and $\Sigma_{t}$, respectively, and we assume that $\Sigma_{t}$ is invertible. We consider deterministic strategies that are self-financing, where we denote by $\theta_{t}$ the strategy for period *t* with each component $\theta_{t,k}$ representing the wealth invested in the kth risky asset at the beginning of period *t*. We model an investor’s preferences by an exponential utility function u(x)=−exp(−γx)/γ, which allows us to incorporate different levels γ of risk aversion. We denote the investor’s wealth at the end of period *t* by $W_{t}$ and the cumulative distribution function (CDF) of $W_{T}$ by $F_{W_{T}}$.

Let us recall the classical expected utility maximization

(1) $$\max_{\theta }E\left[u\left(W_{T}\right)\right],$$

which consists of maximizing the expected utility of the terminal wealth $W_{T}$ over all trading strategies θ. Theorem 1 in Chamberlain (1983) implies that if the returns ${\left(Y_{t}\right)}_{t=1,\cdots ,T}$ are elliptically distributed, (1) is equivalent to the mean-variance portfolio maximization

(2) $$\max_{\theta }\left(E\left[W_{T}\right]-\frac{\gamma }{2}\mathrm{Var}\left(W_{T}\right)\right).$$

It is well known and straightforward to derive that its optimizer is given by

(3) $$\theta_{t}^{*}=\frac{1}{\gamma {\left(1+r_{f}\right)}^{T-t}}\Sigma_{t}^{-1}\left(\mu_{t}-r_{f}𝟙\right),$$

where $𝟙={\left(1,\cdots ,1\right)}^{⊤}$ is a column vector of ones.

**Figure 1:**
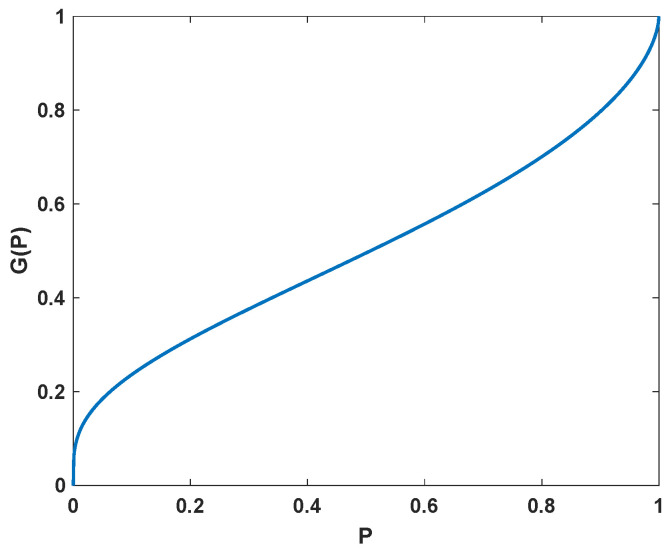
Example of a probability weighting function in the form of $G\left(P\right)=\exp(-{\left(-\beta \ln\left(P\right)\right)}^{\alpha })$, introduced by Prelec (1998), with parameters α=0.6 and β=0.8.

Rather than using classical utility maximization, we consider RDEU, which applies a weighting function *G* to the CDF $F_{W_{T}}$ of the terminal wealth. The weighting function *G* is a continuous and increasing mapping of [0,1] to [0,1] that is inverse *S*-shaped, which allows one to model the investor’s overweighting of unlikely extreme outcomes; see Figure 1 for an example. This means that, instead of (2), we consider the portfolio optimization problem

$$\max_{\theta }E\left[u\left(W_{T}\right)Z\left(F_{W_{T}}\left(W_{T}\right)\right)\right],$$

where *Z* denotes the derivative of *G*. The increased complexity of this portfolio optimization, compared to classical expected utility maximization, does in general not allow for an explicit solution. In light of this, we present two propositions that will enable us to measure the effect of a shift in the risky assets’ expected return on the RDEU value and optimal strategy.

**Proposition** **1.** *Let θ be a strategy with associated terminal wealth $W_{T}$ and consider a shift of the expected returns $\mu_{t}$ in period t to ${\tilde{\mu }}_{t}=\mu_{t}+\kappa_{t}$ for a vector $\kappa_{t}$. Then the log difference of the RDEU is given by*

$$\ln\left(\frac{E[u\left({\tilde{W}}_{T}\right)Z\left(F_{{\tilde{W}}_{T}}\left({\tilde{W}}_{T}\right)\right)]}{E[u\left(W_{T}\right)Z\left(F_{W_{T}}\left(W_{T}\right)\right)]}\right)=-\gamma {\left(1+r_{f}\right)}^{T-t}\theta_{t}^{⊤}\kappa_{t},$$

*where ${\tilde{W}}_{T}$ is the wealth under the shifted expected returns and with the same strategy θ.*

While Proposition 1 demonstrates the impact of a shift in the expected returns on the RDEU, the next result shows how the optimal strategy is affected.

**Proposition** **2.** Assume that the wealth $W_{T}$ is approximately normally distributed and that the weighting function G has an inflection point at approximately 1/2. Let $\theta^{*}$ and ${\tilde{\theta }}^{*}$ be the optimal strategies of the original problem and the problem with shifted time-t expected returns ${\tilde{\mu }}_{t}=\mu_{t}+\kappa_{t}$ for a vector $\kappa_{t}$. Then the change in the optimal strategy is approximately given by

(4) $${\tilde{\theta }}_{t}^{*}-\theta_{t}^{*}\approx \frac{1-2b}{\gamma {\left(1+r_{f}\right)}^{T-t}}\Sigma_{t}^{-1}\kappa_{t},$$

*where $b=Z^{\prime \prime }\left(1/2\right)/\left(4\pi Z(1/2)\right)$.*

The assumption of the weighting function *G* having an inflection point at approximately 1/2 is natural, since *G* is inverse *S*-shaped and maps [0,1] to [0,1]; compare Figure 1.

The approximation (4) tells us how the optimal strategy changes when the expected return vector changes. In the case where the investor’s probabilities are not distorted, the function *G* is the identity function, its derivative *Z* is equal to 1, and $Z^{\prime \prime }=0$ so that b=0 and (4) simplifies to $\frac{1}{\gamma {\left(1+r_{f}\right)}^{T-t}}\Sigma_{t}^{-1}\kappa_{t}$. This is the same as how the optimizer (3) in the classical expected utility maximization is affected under changes of the expected returns. The approximation (4) is more general in that it allows us to incorporate the investor’s biases in their probabilities through the parameter *b*.

The approximation (4) is exact when the distribution of $W_{T}$ is normal and the weighting function takes the form $G\left(P\right)=\Phi (\alpha \Phi^{-1}\left(P\right))$, where Φ is the CDF of the standard normal distribution. With this choice of weighting function, the coefficient 1−2b becomes $\alpha^{2}$. In this special case, the optimal strategy is similar to the example of Section 5 in Hu et al. (2021), who consider equilibrium strategies in a continuous-time model.

## 3. Quantitative Analysis

### 3.1. Estimated Change in ETF and MF Investment

To estimate the benefits that ETF investors receive from the tax loophole, we consider two scenarios from the perspective of a withdrawing ETF investor, where the investor has held their investment for *T* years. In the first scenario the loophole is freely used by the ETF, and in the second scenario the loophole is closed. We assume that the ETF has an annual return of $\mu_{l}$ when using the loophole and an annual return of $\mu_{c}$ when the loophole is closed. The return $\mu_{c}$ is determined by applying a capital gains tax rate, denoted by $r_{t}$, to the ETF return excluding dividends. Setting the dividend rate to be *d*, we find $\mu_{c}=\left(1-r_{t}\right)\left(\mu_{l}-d\right)+d$. If $r_{t}<0$, then $\mu_{c}<\mu_{l}$ and it is clear to see that there are benefits received by taking advantage of the loophole, that in turn compound with an investor’s holding period.

In the scenario where the ETF used the tax loophole and paid no capital gains tax, we assume that the withdrawing investor must pay the tax rate of $r_{t}$ on their capital gains, as this is the corresponding tax rate that would have been applied to the yearly capital gains of the ETF to the investor.^1^ The annualized return for the withdrawing investor after capital gains tax has been applied is given by ${\left({\left(1+\mu_{l}\right)}^{T}-\left({\left(1+\mu_{l}-d\right)}^{T}-1\right)r_{t}\right)}^{\frac{1}{T}}-1$. With an analogous argument, we determine the annualized return after capital gains tax in the scenario where the loophole has been closed as ${\left({\left(1+\mu_{c}\right)}^{T}-\left({\left(1+\mu_{c}-d\right)}^{T}-1\right)r_{c}\right)}^{\frac{1}{T}}-1$, where $r_{c}$ represents the tax rate on capital gains in addition to the yearly capital gains tax. To determine the extent to which ETF investors benefit from the loophole, we compute the difference between the two scenarios. We denote the annualized ETF return benefits received from the tax loophole usage by $\tau_{T}$, and we compute $\tau_{T}$ as

(5) $$\tau_{T}={\left({\left(1+\mu_{l}\right)}^{T}-\left({\left(1+\mu_{l}-d\right)}^{T}-1\right)r_{t}\right)}^{\frac{1}{T}}-{\left({\left(1+\mu_{c}\right)}^{T}-\left({\left(1+\mu_{c}-d\right)}^{T}-1\right)r_{c}\right)}^{\frac{1}{T}}.$$

Using (5), we construct the vector $\kappa_{t}$ from Proposition 2, for all *t*, as $\kappa_{t}={\left[-\tau_{T},0\right]}^{⊤}$. Here, we have set component one of $\kappa_{t}$ to represent the change in an ETF’s expected return and component two of $\kappa_{t}$ to represent an MF’s change in expected return. We retain this setting for the remainder of this paper.

We define $\sigma_{1}^{2}$ to be the variance of the ETF’s returns, $\sigma_{2}^{2}$ the variance of the MF’s returns, and ρ to be the correlation between the two funds’ returns. For simplicity, we assume the variances and correlation to be constant across holding periods. Analyzing (4) in this two dimensional case with change vector $\kappa_{t}$, we find the optimal weight change is approximated by

(6) $${\tilde{\theta }}_{t}^{*}-\theta_{t}^{*}\approx \frac{1-2b}{\gamma {\left(1+r_{f}\right)}^{T-t}}\frac{1}{\left|\Sigma \right|}{\left[-\tau_{T}\sigma_{2}^{2},\;\tau_{T}\rho \sigma_{1}\sigma_{2}\right]}^{⊤},$$

where |Σ| represents the determinant of the covariance matrix and is equal to ${\left(\sigma_{1}\sigma_{2}\right)}^{2}-{\left(\rho \sigma_{1}\sigma_{2}\right)}^{2}$. We note that as the covariance matrix is positive semi-definite, we have |Σ|≥0. We analyze various sensitivities of (6) in Section 3.2.

Next we perform an analysis using reasonable realistic and estimated parameters. For our choice of $r_{t}$ from (5), we note that in 2018, the investors in 400 U.S. equity ETFs avoided paying tax on more than 211 billion USD in gains as a result of tax loophole, which is approximately 23 billion USD in taxes (Mider et al. 2019). This equates to an approximate tax rate of 10.9%, and we set $r_{t}=0.109$. For the dividend rate *d*, we set this equal to 2.5%, the approximate average dividend yield of the S&P 500 over the past 40 years.^2^ We set the risk-free interest rate equal to zero. We also assume, for simplification, that the investor has paid all their capital gains taxes during the holding period of their investment, and as such we set $r_{c}$ to zero.

We conduct our analysis for multiple holding periods and for multiple expected ETF returns. We assume each holding period is equivalent to one period in our model. We choose holding periods between two and 15 years, and choose the expected ETF return $\mu_{l}$ in the range from 6% to 12%. We assume that the MF and ETF are highly correlated, and set their correlation coefficient ρ equal to 0.9. We set the ETF’s and MF’s return standard deviation equal to 15% and 16.5%, respectively. Both the standard deviations and the correlation were chosen in accordance to sample parameters for active and passive funds determined by Polkovnichenko et al. (2019). For the risk aversion parameter, we choose γ=5, and we set the initial wealth equal to one. We use the Prelec weighting function of the form $G\left(P\right)=\exp(-{\left(-\beta \ln\left(P\right)\right)}^{\alpha })$, introduced by Prelec (1998). Polkovnichenko et al. (2019) have shown that this weighting function can be used to accurately represent investor portfolios containing active and passive funds. For the parameters of the weighting function, we set α=0.9 and β=0.95, which are the approximate median values of distortion that represent an accurate level of observed investor bias in the market (Polkovnichenko and Zhao 2013).

As expected, we find from Table 1 that the optimal wealth allocated to the ETF decreases as we decrease its tax efficiency by closing the loophole. Consequentially, more capital is invested into the MF. This relation, however, is not one-to-one, and the remaining capital from the ETF that does not flow into the MF is allocated to the risk-free asset. Table 1 highlights the strong effect of the holding period. As the holding period is increased, so too is the impact of the tax loophole closure to a significant extent, as the benefits received from the loophole are compounding. Additionally, for ETFs with a higher expected return, closing the loophole has a significantly larger impact on the flow of investor capital from ETFs to MFs. These results are consistent to those of Moussawi et al. (2021), as we also find a non-trivial amount of capital will flow from ETFs to MFs in the event the loophole is closed. This analysis demonstrates the influence that the ETF tax loophole currently has on investor portfolios, and the importance that tax legislation as a whole has on asset allocation.

From this application, we can extrapolate an additional result in regards to the proposed tax plan of U.S. President Biden and its potential ramifications for ETFs and MFs. The Biden administration considers raising the capital gains tax from 20% to 39.6% for high-income-earning investors (Davison 2021). In the event the ETF tax loophole remains open and this tax plan becomes legislation, the comparative advantage ETFs have over MFs due to the tax loophole will increase. MF investors affected by the tax plan would need to pay higher capital gains taxes in the relevant tax year while ETF investors could defer them to a future, tax efficient date. This likely would result in further flows of capital into ETFs from MFs and other funds.

### 3.2. Sensitivities

**Figure 2:**
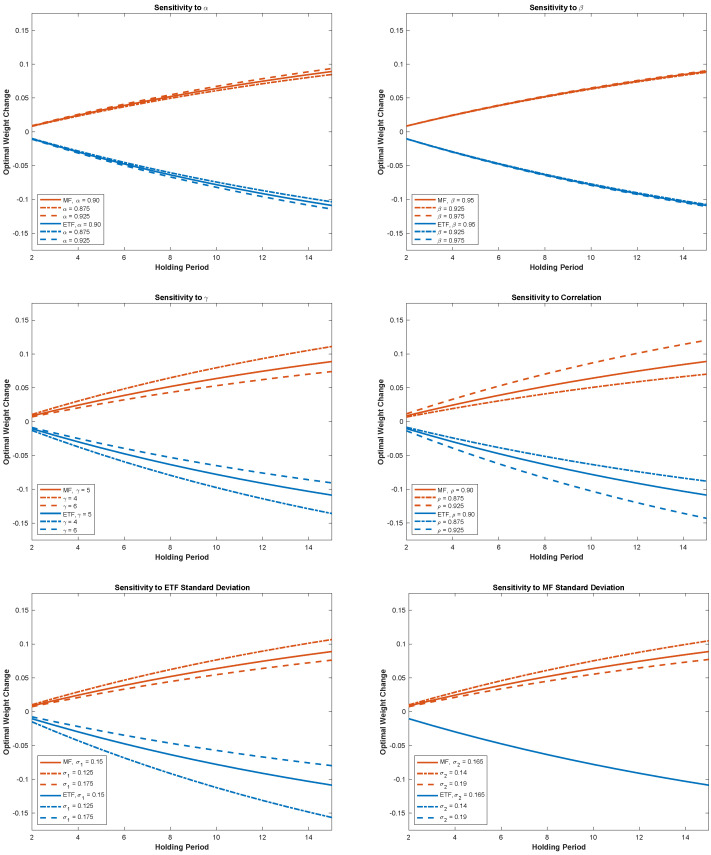

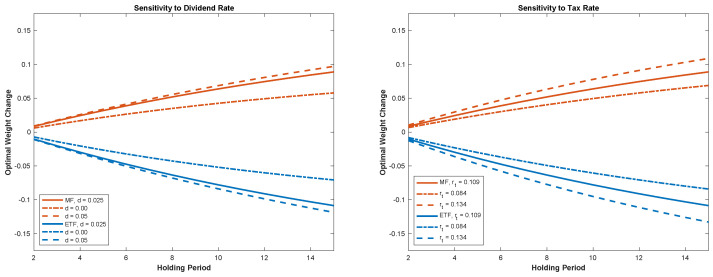
Sensitivity of funds’ optimal weight change given parameter changes.

To further validate our results, we investigate their robustness in a sensitivity analysis. Using the same parameter values as in Section 3.1 to generate Table 1, with the exception of fixing $\mu_{l}$ at 0.08, we now analyze how variations in parameter values impact the optimal weight change described by (6). The results of the sensitivity analysis are similar for values of $\mu_{l}$ other than 0.08. For the parameters α, β, ρ, $\sigma_{1}$, $\sigma_{2}$, *d*, and $r_{t}$, we perturb the original parameter by ±0.025 and re-compute the change in optimal weight for the ETF and MF. For γ, we choose to use ±1. Altering a single parameter at a time, we display our results in Figure 2.

First, we analyze how changes in α and β, the weighting function parameters, impact the change in optimal strategy. We see that changes in either of these parameters result in relatively small changes in strategy, and reducing these parameters decreases the absolute weight change. As both of these parameters increase to one, the probability distortion reduces and we approach the optimal strategy in the classical expected utility maximization. The risk aversion parameter γ, which appears only as a divisor in (6), simply scales the optimal strategy change. Changes in ρ, the correlation between the ETF and MF returns, result in a large change in optimal strategy, and these changes grow with the correlation value. This is because the ρ-dependent terms in (6) can be written as

(7) $$\frac{1}{\left|\Sigma \right|}{\left[-\tau_{T}\sigma_{2}^{2},\;\tau_{T}\rho \sigma_{1}\sigma_{2}\right]}^{⊤}=\frac{1}{1-\rho^{2}}{\left[-\frac{\tau_{T}}{\sigma_{1}^{2}},\;\frac{\tau_{T}\rho }{\sigma_{1}\sigma_{2}}\right]}^{⊤},$$

which shows that the dependence on ρ is proportional to $\frac{1}{1-\rho^{2}}$ for the ETF investment and proportional to $\frac{\rho }{1-\rho^{2}}$ for the MF investment. Analyzing changes in the ETF’s standard deviation, we see that this creates an unequal change between the MF and ETF optimal weights, as shown by the band widths in the figure, while a change in the MF standard deviation only impacts the MF’s optimal weight. Both observations follow directly from (7). Finally, we see that changes in the dividend rate and applied tax rate $r_{t}$ impact both funds’ weights symmetrically.

Overall, we see from this robustness investigation that changes in ρ and the ETF’s standard deviation have the greatest impact on the results given the range of ±0.025, while the least sensitive parameters given our interval are the weighting function parameters α and β, as well as the MF’s standard deviation in the case of the ETF investment. The range of the optimal strategy grows with the holding period for all parameters, which is to be expected given the compounding nature of (6).

## 4. Conclusions

This paper adds to the increasing discussion regarding the ETF tax loophole, by conducting a quantitative analysis of its impact on investor portfolios. As we are dealing with active MFs and passive ETFs, we had to take into account the inherent investor bias between these two types of funds and to do so, we used an RDEU model. Under exponential utility preferences, we can exactly measure the log difference of the RDEU values given an additive shift in the expected asset returns. We are further able to approximate how an investor’s optimal portfolio would change given this additive shift.

By analyzing two scenarios of ETF investors, we can estimate the benefits that investors receive due to the tax loophole, and how their portfolio would change in the event this loophole is closed. The application of the approximation formula allows us to highlight how this estimation depends on the investment horizon and the expected asset returns. Confirming the results of Moussawi et al. (2021), we find a non-trivial amount of an investor’s capital would flow into MFs from ETFs if the loophole is closed. To validate the robustness of our results, we performed sensitivity tests, which highlight the importance of the ETF’s standard deviation and the correlation between the ETF and MF.

Several related areas could be explored in future research. As this paper is based on a discrete-time financial market with *T* trading periods, a similar analysis in a continuous-time model could be interesting. While continuous-time RDEU models are generally time-inconsistent, Hu et al. (2021) are able to characterize RDEU in a continuous-time setting. A further study could deal with how tax revenues will change over time if the tax loophole is closed, since we observed that investors will adjust their strategies in such a scenario. If the tax loophole is indeed closed, an empirical analysis could investigate the flows between ETFs and MFs, comparing them with our results.

## Figures and Tables

**Table 1:** Optimal investment change vector for a portfolio containing an ETF and MF as a function of the investment horizon *T* and the expected ETF return $\mu_{l}$.

	$\mu_{l}$	6%	8%	10%	12%
*T*					
2 years		[−0.006,0.005]	[−0.010,0.009]	[−0.017,0.014]	[−0.024,0.019]
3 years		[−0.011,0.009]	[−0.020,0.017]	[−0.032,0.026]	[−0.049,0.038]
4 years		[−0.016,0.013]	[−0.030,0.024]	[−0.047,0.038]	[−0.067,0.055]
5 years		[−0.021,0.017]	[−0.039,0.032]	[−0.061,0.050]	[−0.086,0.070]
10 years		[−0.043,0.035]	[−0.078,0.064]	[−0.119,0.098]	[−0.166,0.136]
15 years		[−0.061,0.050]	[−0.109,0.089]	[−0.163,0.134]	[−0.223,0.183]

## Data Availability

Data is contained within the article.

## Notes

1 This tax rate may be lower than $r_{t}$, as the investor may decide to realize their gains in a year where they have low income. For simplicity, we assume that it is equivalent.

2 Historical dividend yields are available at https://www.multpl.com (accessed on 28 April 2022).

## Abbreviations

The following abbreviations are used in this manuscript:

CDF	cumulative distribution function
ETF	exchange-traded fund
MF	mutual fund
RDEU	rank-dependent expected utility

## Appendix A. Proofs

**Proof of Proposition** **1.** We first note that ${\tilde{W}}_{T}=W_{T}+{\left(1+r_{f}\right)}^{T-t}\theta_{t}^{⊤}\kappa_{t}$. Let $F_{W_{T}}$ and $F_{{\tilde{W}}_{T}}$ be the CDFs of $W_{T}$ and ${\tilde{W}}_{T}$, respectively. Then, we have the relation

$$\begin{array}{cc} F_{{\tilde{W}}_{T}}\left({\tilde{W}}_{T}\right)\; & =P\left({\tilde{W}}_{T}\leq x\right){|}_{x={\tilde{W}}_{T}}\\ \; & =P\left(W_{T}+{\left(1+r_{f}\right)}^{T-t}\theta_{t}^{⊤}\kappa_{t}\leq x\right){|}_{x=W_{T}+{\left(1+r_{f}\right)}^{T-t}\theta_{t}^{⊤}\kappa_{t}}\\ \; & =P\left(W_{T}\leq x-{\left(1+r_{f}\right)}^{T-t}\theta_{t}^{⊤}\kappa_{t}\right){|}_{x-{\left(1+r_{f}\right)}^{T-t}\theta_{t}^{⊤}\kappa_{t}=W_{T}}\\ \; & =P\left(W_{T}\leq y\right){|}_{y=W_{T}}\\ \; & =F_{W_{T}}\left(W_{T}\right). \end{array}$$

Thus, for any probability weighting function *G* with derivative *Z*, we obtain $Z\left(F_{{\tilde{W}}_{T}}\left({\tilde{W}}_{T}\right)\right)=Z\left(F_{W_{T}}\left(W_{T}\right)\right)$. Because of the exponential utility function, we have the relation

$$u\left({\tilde{W}}_{T}\right)=u\left(W_{T}+{\left(1+r_{f}\right)}^{T-t}\theta_{t}^{⊤}\kappa_{t}\right)=e^{-\gamma {\left(1+r_{f}\right)}^{T-t}\theta_{t}^{⊤}\kappa_{t}}u\left(W_{T}\right)$$

so that

(A1) $$E\left[u\left({\tilde{W}}_{T}\right)Z\left(F_{{\tilde{W}}_{T}}\left({\tilde{W}}_{T}\right)\right)\right]=e^{-\gamma {\left(1+r_{f}\right)}^{T-t}\theta_{t}^{⊤}\kappa_{t}}E\left[u\left(W_{T}\right)Z\left(F_{W_{T}}\left(W_{T}\right)\right)\right],$$

which concludes the proof by taking logarithms. □

To prove Proposition 2, we fist show the following lemma.

**Lemma** **A1.**
*Assume that the RDEU is of the form*

(A2)
$$E\left[u\left(W_{T}\right)Z\left(F_{W_{T}}\left(W_{T}\right)\right)\right]=c\;\exp\left(\frac{1}{2}\sum_{s=1}^{T}\theta_{s}^{⊤}A_{s}\theta_{s}+\sum_{s=1}^{T}\theta_{s}^{⊤}v_{s}\right)$$

*for symmetric and positive definite matrices $A_{s}$ and vectors $v_{s}$. Let $\theta^{*}$ and ${\tilde{\theta }}^{*}$ be the optimal strategies of the original problem and the problem with shifted time-t expected returns ${\tilde{\mu }}_{t}=\mu_{t}+\kappa_{t}$ for a vector $\kappa_{t}$. Then the change in the optimal strategy is given by ${\tilde{\theta }}_{t}^{*}-\theta_{t}^{*}=\gamma {\left(1+r_{f}\right)}^{T-t}A_{t}^{-1}\kappa_{t}.$*

**Proof of Lemma** **A1.** Taking in (A2) the first-order conditions with respect to $\theta_{t}$ and using that $A_{t}$ is symmetric and positive definite, we find $\theta_{t}^{*}=-A_{t}^{-1}v_{t}$. Combining (A1) and (A2), we obtain similarly that ${\tilde{\theta }}_{t}^{*}=\gamma {\left(1+r_{f}\right)}^{T-t}A_{t}^{-1}\kappa_{t}-A_{t}^{-1}v_{t}$, hence we deduce ${\tilde{\theta }}_{t}^{*}-\theta_{t}^{*}=\gamma {\left(1+r_{f}\right)}^{T-t}A_{t}^{-1}\kappa_{t}$. □

**Proof of Proposition** **2.** Using that the wealth $W_{T}$ is approximately normally distributed, we have the approximations $F_{W_{T}}\left(E(W_{T})\right)\approx 1/2$, $F_{W_{T}}^{\prime }\left(E(W_{T})\right)\approx 1/\sqrt{2\pi \mathrm{Var}(W_{T})}$, and $F_{W_{T}}^{\prime \prime }\left(E(W_{T})\right)\approx 0$. We centre a second-order Taylor approximation on $Z\left(F_{W_{T}}\left(W_{T}\right)\right)$ about $E(W_{T})$. Using additionally that our weighting function *G* has an inflection point at approximately 1/2, we form the approximation

$$\begin{array}{cc} \; & Z\left(F_{W_{T}}\left(W_{T}\right)\right)\\ \; & \approx Z\left(F_{W_{T}}\left(E\left(W_{T}\right)\right)\right)+Z^{\prime }\left(F_{W_{T}}\left(E\left(W_{T}\right)\right)\right)F_{W_{T}}^{\prime }\left(E(W_{T})\right)\left(W_{T}-E\left(W_{T}\right)\right)\\ & \;+\frac{Z^{\prime }\left(F_{W_{T}}\left(E\left(W_{T}\right)\right)\right)F_{W_{T}}^{\prime \prime }\left(E\left(W_{T}\right)\right)+Z^{\prime \prime }\left(F_{W_{T}}\left(E\left(W_{T}\right)\right)\right){\left(F_{W_{T}}^{\prime }\left(E\left(W_{T}\right)\right)\right)}^{2}}{2}{\left(W_{T}-E\left(W_{T}\right)\right)}^{2}\\ \; & \approx Z\left(1/2\right)+Z^{\prime }\left(1/2\right)F_{W_{T}}^{\prime }\left(E\left(W_{T}\right)\right)\left(W_{T}-E\left(W_{T}\right)\right)\\ & \;+\frac{Z^{\prime }\left(1/2\right)F_{W_{T}}^{\prime \prime }\left(E\left(W_{T}\right)\right)+Z^{\prime \prime }\left(1/2\right){\left(F_{W_{T}}^{\prime }\left(E\left(W_{T}\right)\right)\right)}^{2}}{2}{\left(W_{T}-E\left(W_{T}\right)\right)}^{2}\\ \; & \approx Z\left(1/2\right)+\frac{Z^{\prime \prime }\left(1/2\right)}{4\pi \mathrm{Var}(W_{T})}{\left(W_{T}-E\left(W_{T}\right)\right)}^{2}\\ \; & =Z\left(1/2\right)\left(1+\frac{Z^{\prime \prime }\left(1/2\right)}{Z\left(1/2\right)\;4\pi \mathrm{Var}\left(W_{T}\right)}{\left(W_{T}-E\left(W_{T}\right)\right)}^{2}\right)\\ \; & \approx Z\left(1/2\right)\exp\left(\frac{Z^{\prime \prime }\left(1/2\right)}{Z\left(1/2\right)\;4\pi \mathrm{Var}\left(W_{T}\right)}{\left(W_{T}-E\left(W_{T}\right)\right)}^{2}\right)\\ \; & =a\exp\left(b{\left(\frac{W_{T}-E\left(W_{T}\right)}{\sqrt{\mathrm{Var}(W_{T})}}\right)}^{2}\right), \end{array}$$

where we have set a=Z(1/2), and $b=Z^{\prime \prime }\left(1/2\right)/\left(4\pi Z(1/2)\right)$. To be able to solve for the optimal strategy using this approximation, we further assume b<1/2. Using this Taylor expansion, and assuming an approximately normal distribution on $W_{T}$, we can write

$$\begin{array}{cc} \; & E\left[u\left(W_{T}\right)Z\left(F_{W_{T}}\left(W_{T}\right)\right)\right]\\ \; & =\int_{-\infty }^{\infty }u\left(w\right)Z\left(F_{W_{T}}\left(w\right)\right)F_{W_{T}}^{\prime }\left(w\right)\;dw\\ \; & \approx \frac{-a}{\gamma \sqrt{2\pi \mathrm{Var}(W_{T})}}\int_{-\infty }^{\infty }\exp\left(-\gamma w+b{\left(\frac{w-E(W_{T})}{\sqrt{\mathrm{Var}(W_{T})}}\right)}^{2}-\frac{1}{2}{\left(\frac{w-E(W_{T})}{\sqrt{\mathrm{Var}(W_{T})}}\right)}^{2}\right)\;dw\\ \; & =\frac{-a}{\gamma \sqrt{2\pi \mathrm{Var}(W_{T})}}\int_{-\infty }^{\infty }\exp\left(-\gamma w+\frac{2b-1}{2}{\left(\frac{w-E(W_{T})}{\sqrt{\mathrm{Var}(W_{T})}}\right)}^{2}\right)\;dw\\ \; & =\frac{-a}{\gamma \sqrt{2\pi }}\int_{-\infty }^{\infty }\exp\left(-\gamma \left(E\left(W_{T}\right)+\sqrt{\mathrm{Var}(W_{T})}y\right)-\frac{1-2b}{2}y^{2}\right)\;dy\\ \; & =\frac{-a}{\gamma \sqrt{1-2b}}\exp\left(\frac{\gamma^{2}\mathrm{Var}\left(W_{T}\right)}{2-4b}-\gamma E\left(W_{T}\right)\right)\\ \; & =\frac{-a\;e^{-\gamma W_{0}{\left(1+r_{f}\right)}^{T}}}{\gamma \sqrt{1-2b}}\exp\left(\frac{\gamma^{2}}{2-4b}\sum_{s=1}^{T}{\left(1+r_{f}\right)}^{2T-2s}\theta_{s}^{⊤}\Sigma_{s}\theta_{s}-\gamma \sum_{s=1}^{T}{\left(1+r_{f}\right)}^{T-s}\theta_{s}^{⊤}\left(\mu_{s}-r_{f}𝟙\right)\right)\\ \; & =\frac{-a\;e^{-\gamma W_{0}{\left(1+r_{f}\right)}^{T}}}{\gamma \sqrt{1-2b}}\exp(\frac{1}{2}\sum_{s=1}^{T}\theta_{s}^{⊤}\left(\frac{\gamma^{2}{\left(1+r_{f}\right)}^{2T-2s}}{1-2b}\Sigma_{s}\right)\theta_{s}\\ \; & \;\;\;\;\;\;\;-\sum_{s=1}^{T}\theta_{s}^{⊤}{\left(1+r_{f}\right)}^{T-s}\left(\gamma \mu_{s}-\gamma r_{f}𝟙\right)). \end{array}$$

Therefore, the matrix $A_{t}$ from Lemma A1 is approximately given by $A_{t}\approx \frac{\gamma^{2}{\left(1+r_{f}\right)}^{2T-2t}}{1-2b}\Sigma_{t}$, which is symmetric and positive definite since $\Sigma_{t}$ is symmetric and positive semi-definite as a covariance matrix, and it is positive definite because of the assumed invertibility. Thus, we conclude (4) from Lemma A1. □

[custom]
